# Extracorporeal shock wave therapy for temporomandibular disorders: efficacy on pain, maximal mouth opening, and dysfunction—a meta-analysis of randomized controlled trials

**DOI:** 10.3389/fresc.2026.1883205

**Published:** 2026-07-09

**Authors:** Li Chen, Yuan Luo, Liyue Zhang, Xiaoyi Zhang, Yi Chen, Dong Hu

**Affiliations:** 1 The First People’s Hospital of Neijiang City, Neijiang, China; 2 Southwest Medical University, China; 3 Chengdu University of Traditional Chinese Medicine, Chengdu, China

**Keywords:** craniomandibular joint syndrome, extracorporeal shockwave therapy, meta-analysis, temporomandibular disorders, temporomandibular joint disease

## Abstract

**Objective:**

Temporomandibular disorders (TMD) are common heterogeneous conditions characterized by chronic orofacial pain, limited maximal mouth opening, and impaired masticatory function. Extracorporeal shock wave therapy (ESWT) is a well-established non-invasive rehabilitation intervention, but its efficacy in improving pain, maximal mouth opening (MMO), and dysfunction index (DI) remains controversial. This study aims to evaluate the therapeutic effectiveness of ESWT in patients with TMD through a systematic review and meta-analysis.

**Methods:**

We conducted a systematic search in PubMed, Embase, Web of Science, Cochrane Library, CNKI, Wanfang Database, and VIP Database, with the search period ending in December 2025. Randomized controlled trials (RCTs) comparing ESWT with rehabilitation interventions, sham therapy, medication, or no treatment were included. The main outcome measures were pain intensity (VAS), maximal mouth opening (MMO), and dysfunction index (DI).

**Results:**

A total of 18 studies involving 1,150 participants were included. ESWT showed a significant therapeutic effect on pain reduction (MD = −1.28; 95% CI: [−1.96, −0.61], *Z* = 3.75, *P* < 0.00001, I^2^ = 95%). ESWT significantly increased maximal mouth opening (MD = 3.76; 95% CI: [1.52, 5.99], *Z* = 3.29, *P* = 0.0010, I^2^ = 97%). No significant improvement was observed in dysfunction index (MD = −0.05; 95% CI: [−0.13, 0.03], *Z* = 1.24, *P* = 0.22, I^2^ = 99%). Through subgroup analysis based on pain assessment, we further explored the sources of heterogeneity related to shock number, treatment period, and gender.

**Conclusion:**

The results of this meta-analysis indicate that ESWT has a significant positive effect in alleviating pain and improving maximal mouth opening in patients with TMD. However, because of the existence of heterogeneity and potential risk factors, the therapeutic effect of ESWT needs to be interpreted with caution.

## 1 Introduction

Temporomandibular disorders (TMD) are a common group of musculoskeletal conditions involving the temporomandibular joint, masticatory muscle groups, and associated maxillofacial structures (1). Core clinical characteristics include pain in the temporomandibular joint or masticatory muscle regions, clicking or crepitus during joint movement, restricted mouth opening or mandibular deviation, and accompanying chewing dysfunction, headache, ear discomfort, and in some cases, a sensation of joint catching or locking during mandibular movement (2, 3). Chronic TMD is defined by a disease course exceeding 3 months, recurrent symptoms, or persistent non-resolution; pain and functional impairments in this subset often exhibit a pattern of continuous or intermittent worsening (4). TMD is a major condition affecting oral health and quality of life globally, with a prevalence of approximately 10% to 15% in the general population (5, 6). It is more common in young and middle-aged individuals, with a significantly higher prevalence in females than in males (7, 8). Beyond impairing basic physiological functions such as chewing, swallowing, and speech, long-term pain associated with TMD may lead to psychological comorbidities including anxiety and depression. These sequelae severely disrupt patients’ daily life, work, and social activities, while also imposing a substantial economic and healthcare burden on family caregivers and healthcare systems (9, 10). Currently, TMD management is primarily conservative, emphasizing multidisciplinary collaborative intervention. Physical therapy constitutes one of the core treatment modalities, widely utilized for pain control and functional improvement. Traditional physical therapy approaches include hot/cold compresses, ultrasound therapy, electrical stimulation, joint mobilization, masticatory muscle relaxation training, and occlusal splint intervention (11, 12). The primary clinical goal is to alleviate pain in the temporomandibular joint and masticatory muscle regions, restore normal mouth opening function, and improve dysfunction, ultimately enhancing patients' quality of life (13).

Extracorporeal shock wave therapy (ESWT) is a non-invasive physical factor treatment modality that uses high-energy sound waves (or pressure waves) as its core medium (14). This therapy preserves skin integrity, penetrating superficial body tissues (e.g., skin, fat, muscle) to deliver mechanical energy precisely to target tissues. Through multiple physical and biological effects—including cavitation, mechanical stimulation, and inflammation modulation—it remodels the local tissue microenvironment, initiating tissue repair and regeneration processes to ultimately achieve therapeutic goals of pain relief and restoration of organ physiological function (15–17). As a key intervention in musculoskeletal rehabilitation, ESWT has gained wide clinical recognition (18, 19). Originating in the 1980s for the treatment of urinary calculi in urology, this technology has gradually expanded to the management of musculoskeletal disorders. Currently, it is recommended by authoritative institutions such as the International Society for Shock Wave Therapy (ISTU) and the World Confederation of Physical Therapy (WCPT) for various conditions, including temporomandibular joint arthritis. Its core treatment principle is to minimize tissue damage while achieving the dual benefits of pain control and functional recovery (20, 21). Numerous clinical studies have confirmed the definite efficacy of ESWT for musculoskeletal diseases, as it effectively alleviates pain symptoms, reduces local inflammatory responses, and promotes the repair of damaged tissues. For temporomandibular joint arthritis specifically, ESWT can significantly improve joint range of motion, reduce pain and discomfort during mouth opening and chewing, and ultimately enhance patients' quality of life (22–24).

As an adjunctive treatment for TMD, extracorporeal shock wave therapy (ESWT) has remained a controversial topic regarding its therapeutic efficacy (25). In recent years, randomized controlled trials (RCTs) investigating the use of ESWT for temporomandibular disorders (TMD) have been increasingly reported (26–28). However, substantial variations exist in patient characteristics, treatment parameters (e.g., energy flux density, number of treatment sessions), follow-up durations, and outcome measurement tools across these studies, resulting in inconsistent conclusions regarding therapeutic effects. Given the limitations of individual studies, this article systematically synthesizes eligible RCT evidence via meta-analysis to objectively and comprehensively evaluate the clinical efficacy of ESWT for TMD, thereby providing high-quality scientific evidence to inform clinical decision-making in TMD rehabilitation.

## 2 Materials and methods

The study preparation and reporting adhered to the methodological standards described in the Cochrane Handbook for Systematic Reviews of Interventions (29) and the Preferred Reporting Items for Systematic Reviews and Meta-Analyses (PRISMA) statement (30).

### 2.1 Search strategy

A systematic electronic literature search was performed across seven databases, namely PubMed, Embase, Web of Science, the Cochrane Library, China National Knowledge Infrastructure (CNKI), VIP Database, and Wanfang Database. The search encompassed all records from the inception of each database to December 2025 with no language restrictions. Guided by the PICOS framework, a combination of topic-specific keywords and their synonyms was adopted to ensure the comprehensive and precise retrieval of relevant studies. The detailed search terms used are as follows: (Extracorporeal shock wave therapy) OR (ESWT) OR (shock wave therapy) OR (extracorporeal pressure wave therapy) AND (Temporomandibular disorders) OR (TMD) OR (temporomandibular joint disease) OR (temporomandibular joint dysfunction).Only studies that met the pre-specified PICOS criteria were eligible for subsequent screening, with the criteria defined as follows: (P) Participants with a confirmed diagnosis of temporomandibular disorders (TMD), irrespective of age, gender, disease duration or severity; (I) Intervention involving extracorporeal shock wave therapy (ESWT), either as a monotherapy or in combination with other therapeutic modalities; (C) Comparison with conventional physical therapy, pharmacological therapy, sham ESWT, or no specific intervention; (O) Core outcome measures including Visual Analogue Scale (VAS) scores for pain intensity, maximal mouth opening (MMO), and temporomandibular joint dysfunction index (DI); (S) Study design of randomized controlled trials (RCTs).

### 2.2 Study selection process

The four authors (LC, YL, XYZ, YC) employed a step—by—step evaluation approach to establish the inclusion criteria. They meticulously examined the titles, abstracts, and full texts of the retrieved studies (in cases of doubt), and subsequently carried out a comprehensive appraisal of the remaining articles in accordance with the pre—defined standards to reach the final inclusion determinations. In instances of disagreement or uncertainty, two authors (LYZ and DH) offered assistance by independently implementing the same evaluation method to settle the disputes. All the literature search records were systematically arranged using EndNote 21 software.

### 2.3 Data extraction

Two independent reviewers (LC and YL) extracted data from each included article, including the following information: authors, year of publication, country/region, participants' demographic characteristics (e.g., mean age, gender distribution), total sample size (with separate sample sizes for the experimental and control groups), detailed ESWT intervention parameters (e.g., energy flux density in mJ/mm^2^, total number of treatment sessions, intervention duration), type of control intervention (e.g., sham ESWT, ultrasound therapy, pharmacotherapy, or no treatment), and reported outcomes (e.g., pre- and post-treatment Visual Analogue Scale [VAS] pain scores, maximal mouth opening [MMO] values, and temporomandibular joint dysfunction index [DI] scores).Any discrepancies between the two reviewers during the data extraction process were resolved by consulting a third arbitrator (LYZ) to reach a consensus.

### 2.4 Intervention and outcomes

Interventions included in this meta-analysis were extracorporeal shock wave therapy (ESWT) regimens administered for the management of temporomandibular disorders (TMD). These protocols were delivered either as monotherapy or combined with conventional physical therapy, pharmacotherapy, or traditional Chinese medicine interventions, with variations in energy flux density, pulse frequency, total impulses, treatment frequency, and overall intervention duration. The decision to provide an initial aggregate estimate across these heterogeneous ESWT protocols is theoretically justified by their shared transdiagnostic objective: relieving pain, restoring mandibular mobility, and improving periarticular tissue function to target the core clinical manifestations of TMD. Despite differences in parameters and adjuvant treatments, all ESWT interventions act on key pathophysiological mechanisms of TMD, including local inflammation, nociceptor sensitization, masticatory muscle hypertonia, and mandibular kinematic disturbance. This initial pooling establishes a global benchmark for ESWT in TMD rehabilitation and provides a necessary baseline for subsequent refined subgroup analyses.

The primary outcomes assessed were pain intensity, maximal mouth opening (MMO), and temporomandibular joint dysfunction index (DI). Pain intensity was measured using the Visual Analogue Scale (VAS), a well-validated instrument with high reliability and responsiveness for assessing orofacial pain in patients with TMD. Maximal mouth opening was quantified as the maximal linear distance between the upper and lower incisors during voluntary mouth opening, a widely accepted gold-standard indicator reflecting mandibular movement capacity and temporomandibular joint function. The dysfunction index provided a comprehensive evaluation of functional impairment, including joint sounds, masticatory disturbance, movement limitation, and daily activity interference, representing a multidimensional measure of TMD-related dysfunction. All outcomes were assessed at baseline and post-intervention using standardized and validated measurement tools, ensuring consistency and comparability across included studies.

### 2.5 Assessment of risk of bias and quality

This review employed the Cochrane Risk of Bias Tool (RoB 2), as recommended by the Cochrane Collaboration, to systematically assess the methodological quality of the included randomized controlled trials. Bias risk was evaluated across five key domains (1): randomization process; (2) deviations from intended interventions; (3) missing outcome data; (4) measurement of the outcome; and (5) selection of the reported result. Each domain was judged as “low risk”, “some concerns”, or “high risk” of bias, in accordance with the RoB 2 framework. Disagreements between the two independent reviewers were resolved through discussion, with arbitration by a third senior reviewer when consensus could not be reached.

### 2.6 Statistical analysis

All statistical analyses were performed using Review Manager 5.4 and Stata 17.0 software. For continuous outcome measures—including Visual Analogue Scale (VAS) pain scores, maximum mouth opening (MMO), and temporomandibular joint dysfunction index (DI)—effect sizes were calculated using the mean difference (MD) or standardized mean difference (SMD), each with a 95% confidence interval (CI). Specifically, the SMD was used for pooled analysis when different measurement tools were employed to assess the same outcome measure. Heterogeneity across studies was comprehensively evaluated using the Cochran's *Q* test and Higgins I² statistic. A fixed-effect model was used for data pooling if heterogeneity was low (*P* > 0.1 and I^2^ < 50%). In contrast, a random-effect model was applied if heterogeneity was high (*P* < 0.01 and I^2^ > 50%), with subgroup analyses conducted to explore potential sources of heterogeneity. Sensitivity analysis was performed using the leave-one-out method to verify the robustness of the meta-analysis results. Statistical significance was set at *P* < 0.05.

### 2.7 Subgroup analysis and sensitivity analysis

To explore potential sources of the observed heterogeneity across included studies, subgroup analyses were conducted for the primary and secondary outcomes based on predefined categorical moderators. Subgroup stratifications were performed according to treatment duration, ESWT application mode (monotherapy vs. combined therapy), and control type (active treatment vs. sham/placebo or no intervention), to identify whether effect sizes differed across these clinical and methodological variables.To verify the stability and reliability of the pooled effect estimates, sensitivity analysis was carried out using the leave-one-out method and by recalculating the overall effects after excluding individual trials with high risk of bias. This approach enabled us to assess the extent to which low-quality studies or single outliers influenced the robustness of the main findings. Finally, publication bias was evaluated qualitatively by visual inspection of funnel plots and quantitatively using Egger's regression test and Begg's test. These assessments were limited to outcomes reported by at least 10 included studies to ensure sufficient statistical power and reliability of the tests.

## 3 Results

### 3.1 Study search results

**Figure 1:**
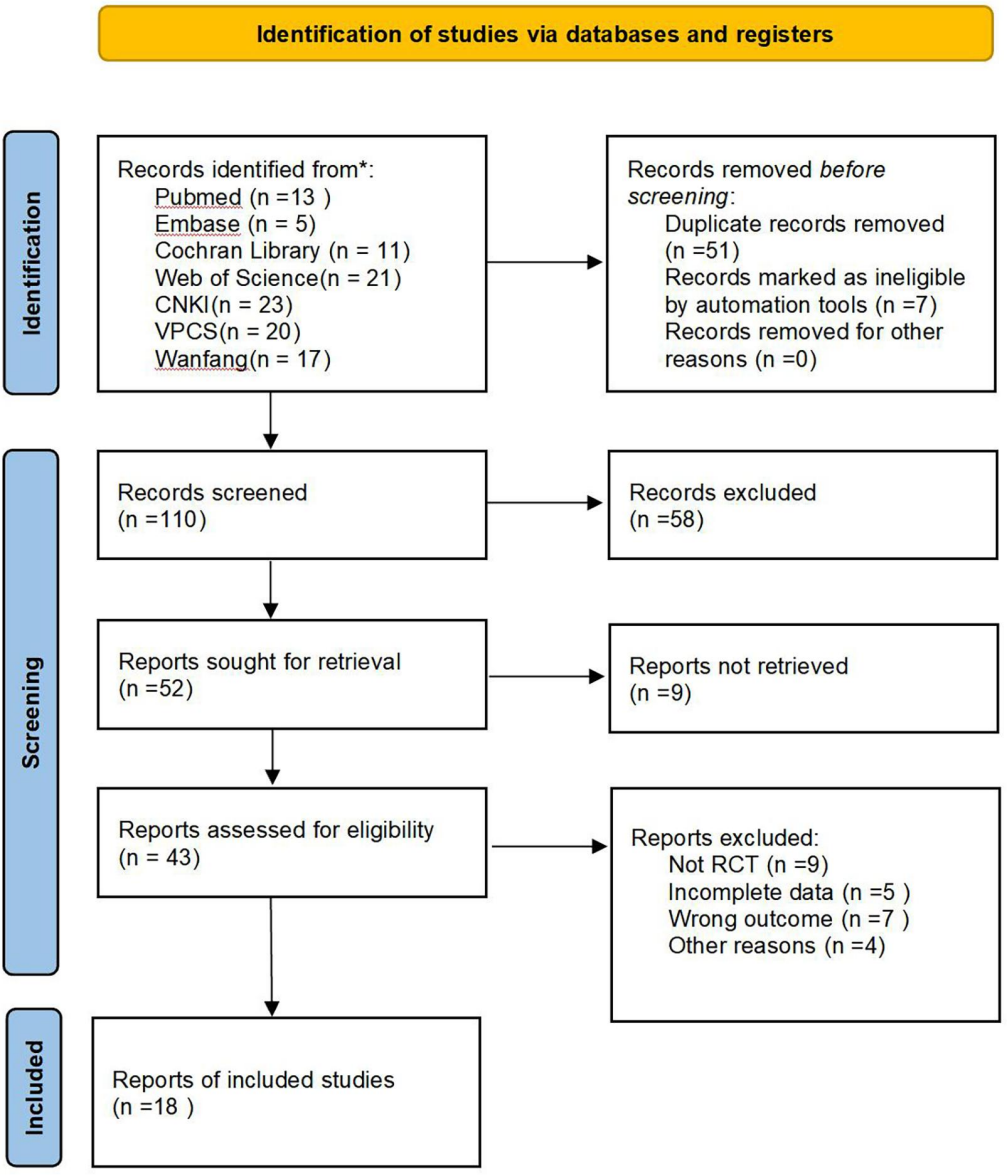
Flow diagram of the systematic literature search..

A systematic search of seven electronic databases identified 110 studies, with the following retrieval counts per database: PubMed (*n* = 13), Embase (*n* = 5), the Cochrane Library (*n* = 11), Web of Science (*n* = 21), CNKI (*n* = 23), VIP Database (*n* = 20), and Wanfang Database (*n* = 17). Prior to formal screening, 51 duplicate studies were removed, and an additional 7 studies were excluded via automated tool screening for failure to meet basic eligibility criteria. Of the remaining 52 retrievable reports, 9 could not be successfully accessed. The subsequent 43 reports were evaluated against the pre-defined inclusion and exclusion criteria, with 25 studies excluded for specific reasons: 9 were non-randomized controlled trials (non-RCTs), 5 had incomplete outcome data, 7 lacked the required core outcomes (Visual Analogue Scale [VAS] scores, maximum mouth opening [MMO], or temporomandibular joint dysfunction index [DI]), and 4 were excluded for other reasons. Ultimately, 18 studies were included in the meta-analysis (Figure 1).

### 3.2 Study characteristics

The 18 randomized controlled trials (RCTs) included in this meta-analysis involved a total of 1,150 participants, consisting of 509 males and 601 females. The publication years of these eligible studies ranged from 2018 to 2025 (note: the search strategy covered all records from the establishment of each database through December 2025; this time range refers specifically to the publication dates of the included studies). Among these 18 RCTs, 6 were published in English (31–36) and 12 were published in Chinese (37–48).

Regarding intervention strategies, 5 RCTs (31, 35, 36, 42, 44) used ESWT as the sole intervention. 8 additional RCTs (32, 33, 37–40, 46, 47) combined ESWT with other physical therapy modalities, while 3 RCTs (34, 44, 45) administered ESWT in conjunction with pharmacotherapy. The remaining 2 RCTs (43, 48) combined ESWT with traditional Chinese medicine (TCM) therapy.

For outcome measurements, 17 studies (31, 32, 34–48) assessed pain intensity using the Visual Analogue Scale (VAS). 12 studies (31, 34, 36, 37, 39–42, 44, 45, 47, 48) evaluated maximum mouth opening (MMO) of the temporomandibular joint,13 studies (33, 35–38, 40–46, 48) assessed the temporomandibular joint dysfunction index (DI).

The units for the outcome measures are specified as follows: The Visual Analogue Scale (VAS) score for pain intensity is a dimensionless value, ranging from 0 to 10 points (Table 1).

**Table 1:** Core raw data of meta-analysis for extracorporeal shock wave therapy in the treatment of temporomandibular disorders (TMD).

Analysis index	Number of included studies (items)	Number of included patients (cases)	Heterogeneity (I2)	statistic model	Mean difference (MD, 95% CI)	*P*-value
Pain intensity (VAS)	17	1,036	95%	random effects model	−1.28 (−1.96, −0.61)	<0.00001
Maximum mouth opening degree (MMO)	12	823	97%	random effects model	3.76 (1.52, 5.99)	0.0010
Joint Function Disability Index (DI)	13	850	99%	random effects model	−0.05 (−0.13, 0.03)	0.22
The study included a total of	18	1,150 (female509/male 601)	—	—	—	—

1. A total of 18 randomized controlled trials (RCTs) were included, with publication time from 2018 to 2025 (6 in English, 12 in Chinese), involving 1,150 patients (509 males, 601 females). 2. VAS = Visual Analogue Scale, CI = confidence interval. 3. Statistical analysis was performed with Review Manager 5.4 and Stata 17.0 software.

Pain intensity (VAS score): 17 included studies, 1,036 patients, heterogeneity I2 = 95%, random-effects model, mean difference (MD) = −1.28 (95%CI: −1.96, −0.61), *P* < 0.00001. Maximum mouth opening (MMO): 12 included studies, 823 patients, heterogeneity I^2^ = 97%, random-effects model, mean difference (MD) = 3.76 (95%CI: 1.52, 5.99), *P* = 0.0010. Temporomandibular joint dysfunction index (DI): 13 included studies, 850 patients, heterogeneity I2 = 99%, random-effects model, mean difference (MD) = −0.05 (95%CI: −0.13, 0.03), *P* = 0.22.

### 3.3 Risk of bias assessment results

**Figure 2:**
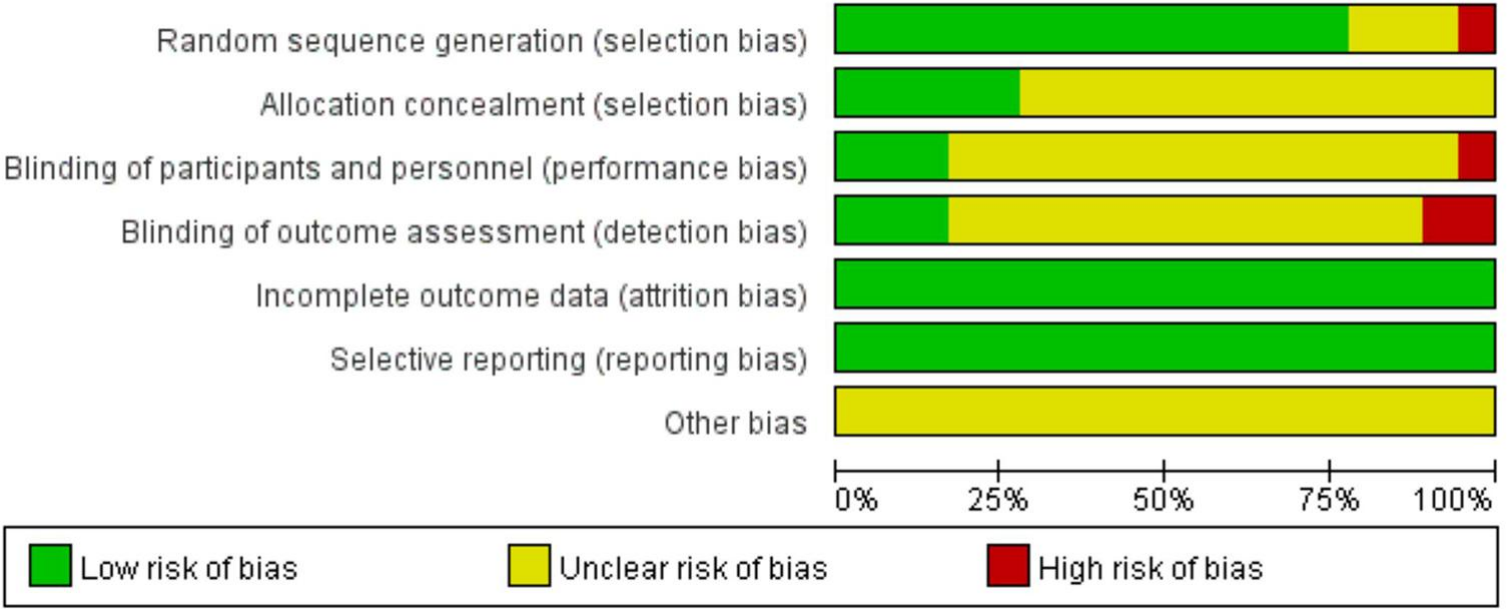
Risk of bias graph.

**Figure 3:**
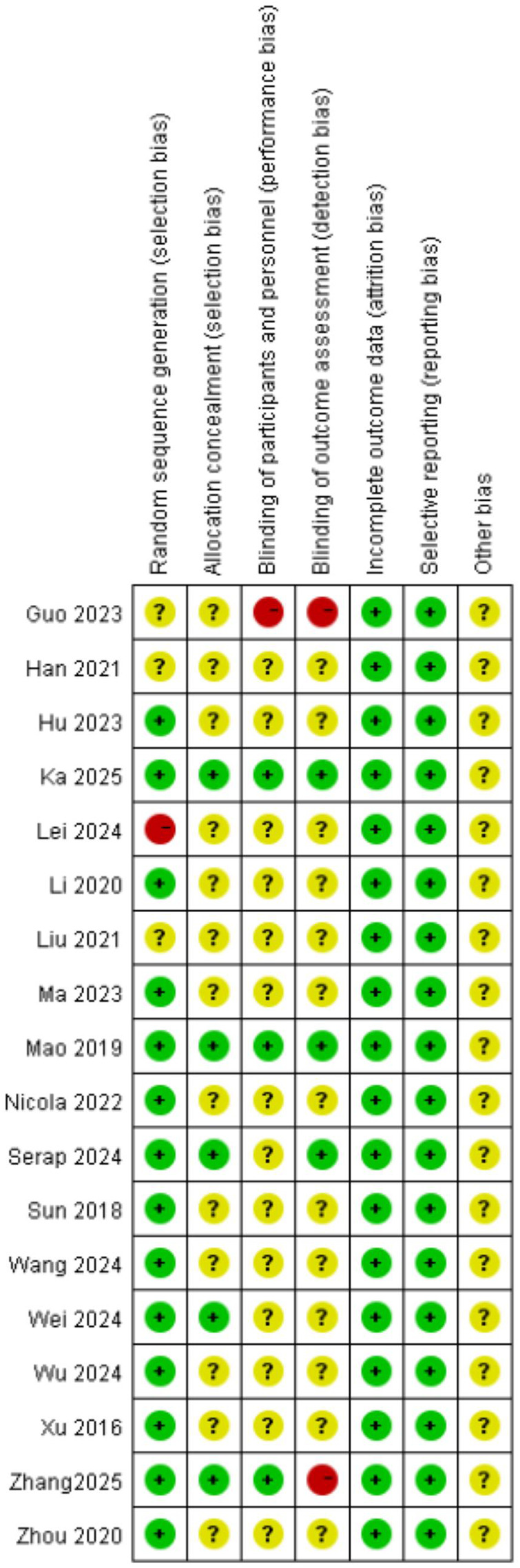
Risk of bias summary.

The risk of bias for included randomized controlled trials (RCTs) was assessed in accordance with the *Systematic Review of Intervention Measures Manual*, covering seven specific domains. Among the 18 included studies, 3 (35, 42, 44) exhibited a relatively high risk of bias in at least one domain. In contrast, 15 studies (31–34, 36–41, 43, 45–48) showed no high risk of bias in any domain, though the risk of bias in at least one domain remained unclear for this subgroup.Overall, the risk of bias rating for the included studies was categorized as “unclear,” primarily due to insufficient detailed reporting of allocation concealment methods (Figures 2, 3).

### 3.4 Outcome

#### 3.4.1 Pain assessment

**Figure 4:**
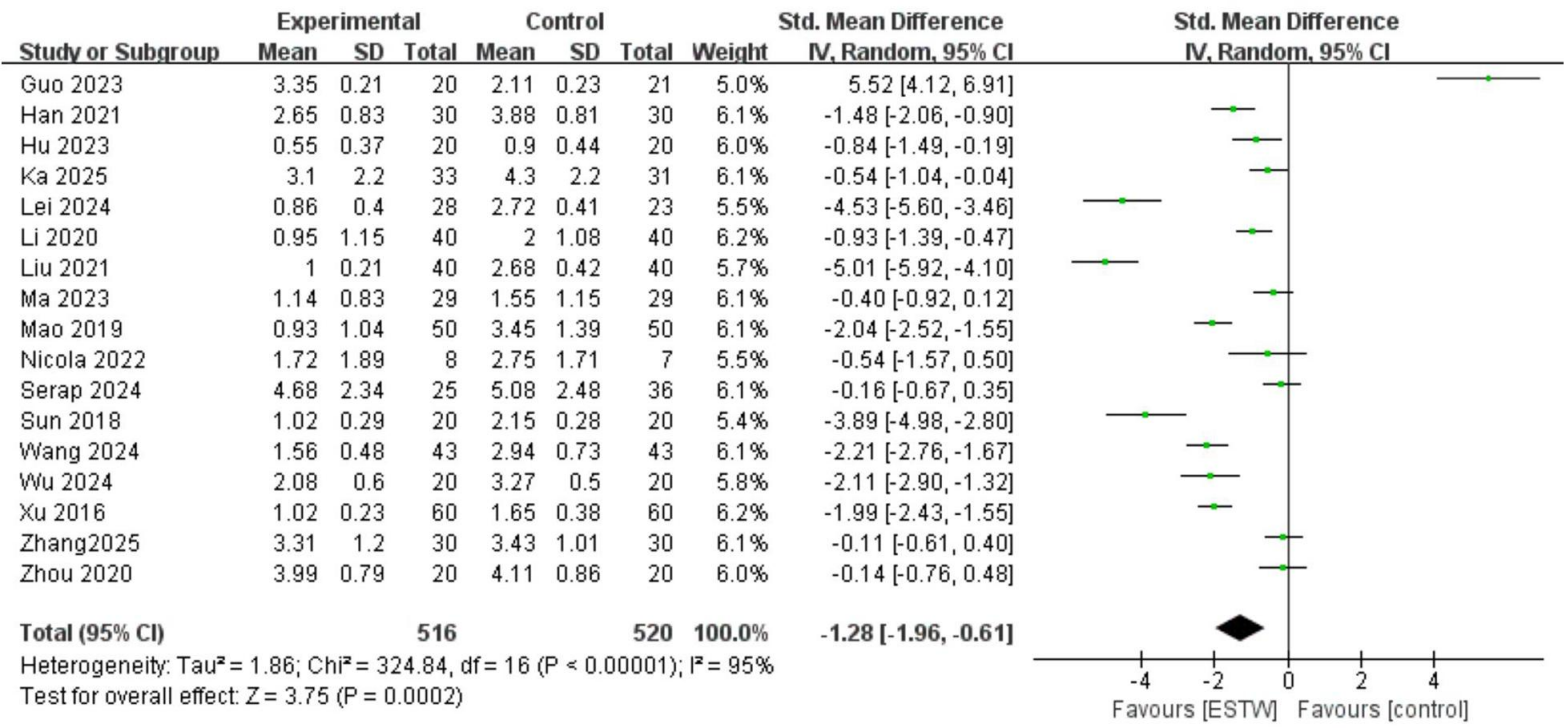
Forest plot of the effect of ESWT on pain.

Pain outcomes were assessed using the Visual Analogue Scale (VAS) or the Numerical Rating Scale (NRS). The meta-analysis included 17 studies involving 1,036 patients, with lower scores indicating milder pain severity. Significant heterogeneity was observed among the included studies (I^2^ = 95%, *P* < 0.00001). Consequently, a random-effects model was employed for the analysis. The results demonstrated a significantly greater reduction in pain in the ESWT group compared to the control group (MD = −1.28; 95% CI: [−1.96, −0.61], Z = 3.75, *P* < 0.00001; Figure 4). To explore potential sources of heterogeneity, subgroup analyses were performed based on number of shocks, treatment period, and gender (Table 2). Significant pain relief was observed in all subgroups.However, only intervention period yielded a significant subgroup difference (*P* < 0.05).

**Table 2:** Effects of ESWT on pain scale improvement: subgroup analysis based on 3 variables.

Variables	Group (n)	Participant (n)	MD [95%Cl]	*P*	1^2^%	Sub-group difference	
						X^2^	*p*
Number of shocks							
≤1,500	6	403	−0.97[−1.80.−0.13]	0.020	91%	0.05	0.82
>1,500	8	501	−1.07[−1.46.−0.69]	≤0.00001	95%		
Period							
≤2WK	14	906	−0.79[−1.38, −0.20]	0.0090	99%	8.71	0.003
≥4WK	3	130	−1.79[−2.09.−1.49]	<0.00001	5%		
Gender							
Female>Male	7	461	−0.82–1.28.−0.36]	<0.00001	96%	0.07	0.79
Male>Female	10	575	−0.97–1.94.0.00]	<0.00001	99%		

MD, mean difference; CI, confidence interval.

#### 3.4.2 Evaluation of mouth opening degree

**Figure 5:**
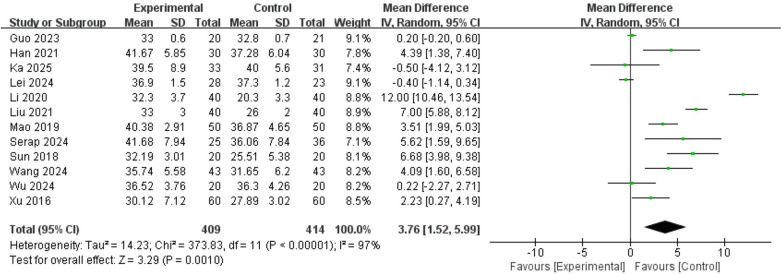
The forest plot shows the therapeutic effect of ESWT on the change in mouth opening angle.

A total of 12 studies were conducted to evaluate the mouth opening degree of patients suffering from temporomandibular joint disorders, encompassing 823 patients. Heterogeneity analysis revealed significant disparities among the studies (I^2^ = 97%). After being analyzed using the random—effects model, the results demonstrated that the improvement in the mouth opening degree of the ESWT group was superior to that of the control group, and the results were statistically significant (MD = 3.76; 95% CI: [1.52, 5.99], *Z* = 3.29, *P* = 0.0010), (Figure 5).

#### 3.4.3 Functional impairment index score

**Figure 6:**
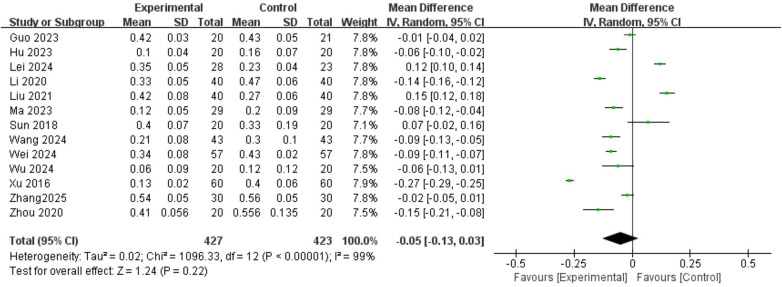
The forest plot shows the efficacy of ESWT in altering functional disorders.

Thirteen studies were included in the meta-analysis of the temporomandibular joint dysfunction index, encompassing a total of 850 participants. Heterogeneity assessment revealed substantial between-study heterogeneity (I^2^ = 99%). Given this high heterogeneity, a random-effects model—rather than a fixed-effects model—was deemed statistically appropriate for pooled effect estimation. Under the random-effects model, no statistically significant difference was observed in dysfunction improvement between the experimental and control groups (mean difference [MD] = −0.05; 95% confidence interval [CI]: −0.13, 0.03; *Z* = 1.24; *p* = 0.22), (Figure 6).

#### 3.4.4 Sensitivity analysis

**Figure 7:**
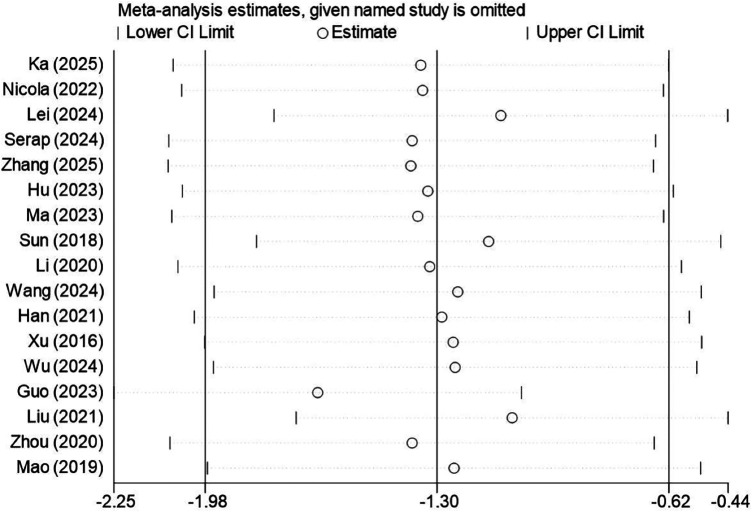
Sensitivity analysis of the pain conditions caused by temporomandibular joint disorders in the two groups of patients.

Sensitivity analysis employing the leave-one-out approach demonstrated the robustness of the pooled effect estimates. Sequential exclusion of each individual study yielded consistent findings: the 95% confidence intervals for the visual analog scale (VAS) and maximum mouth opening (MMO) outcomes remained entirely outside the null value (i.e., did not include zero), whereas the dysfunction index (DI) results remained statistically non-significant across all iterations, with no meaningful shift in effect magnitude or direction. Collectively, these findings confirm the stability and reliability of the meta-analytic conclusions,as illustrated in Figure 7.

### 3.5 Publication bias

**Figure 8:**
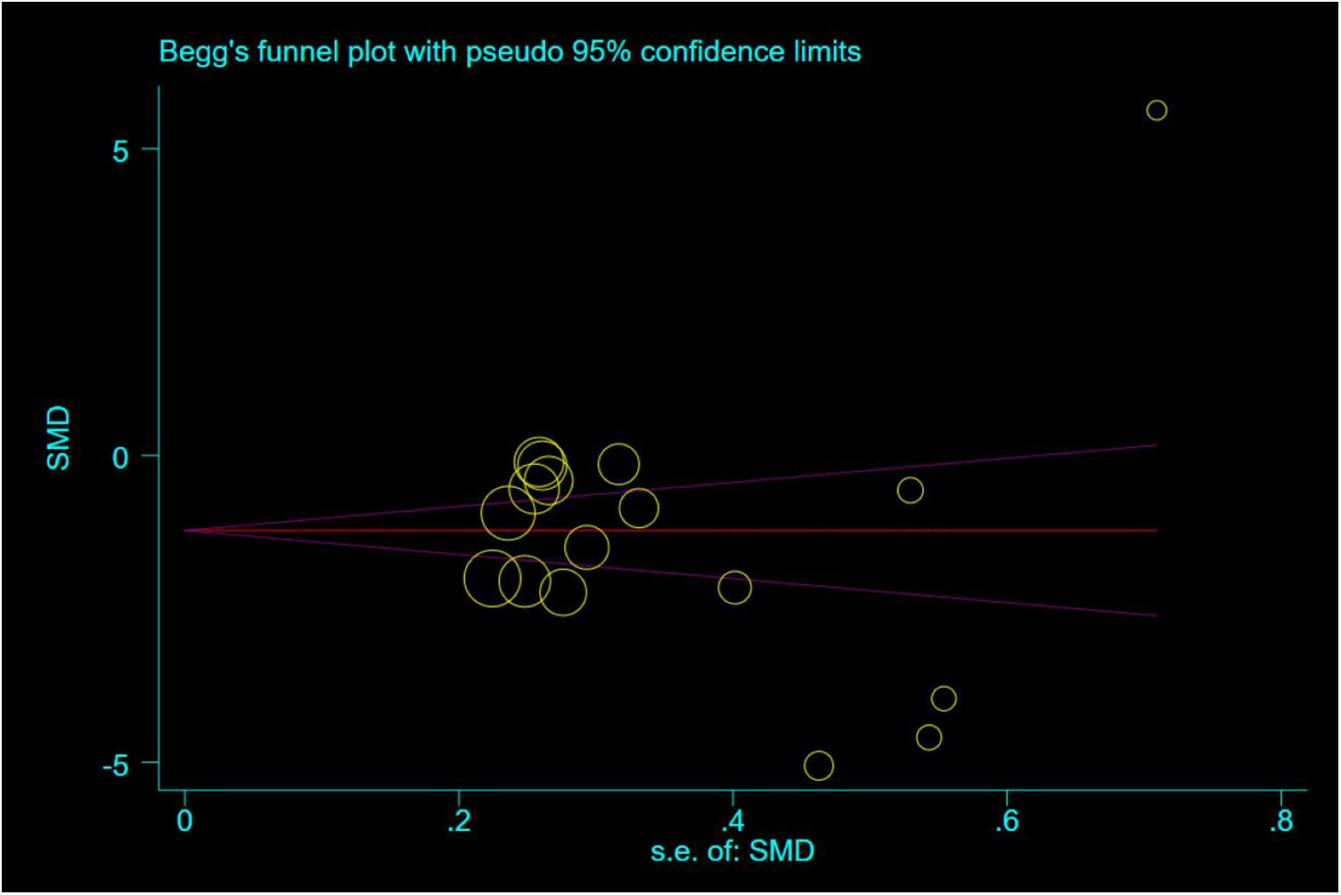
Begg's funnel plot with pseudo 95% confidence limits.

**Figure 9:**
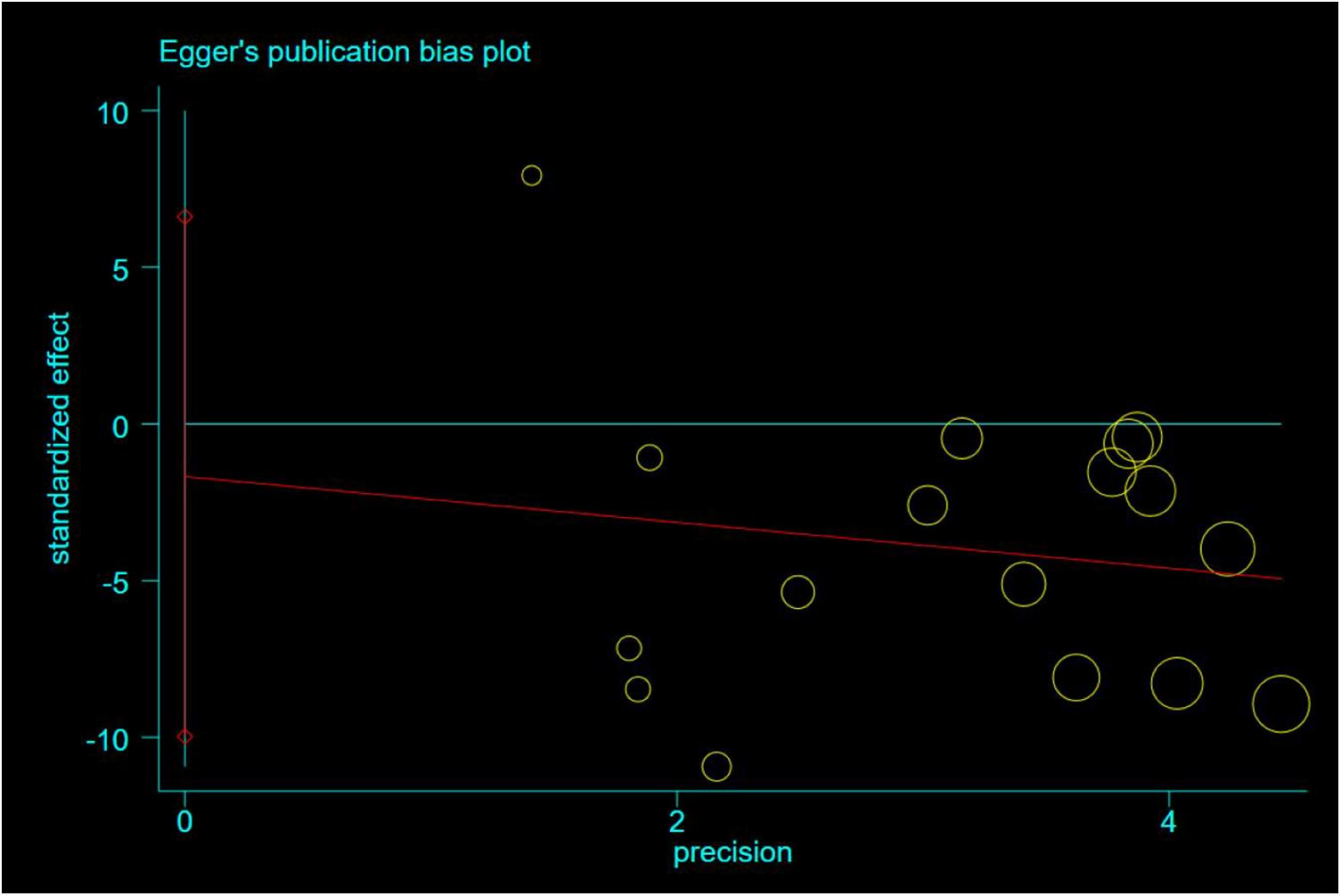
Egger's publication bias plot.

Publication bias was assessed using Egger's and Begg's tests for the primary outcome of pain relief in temporomandibular disorders. Begg's funnel plot showed no obvious asymmetry, and both tests indicated no significant publication bias (Egger's test: *p* = 0.672; Begg's test: *p* = 0.537), (Figures 8, 9).

## 4 Discussion

Significant pain reduction was observed following extracorporeal shock wave therapy (ESWT) for temporomandibular joint disorders (TMD), with a mean difference (MD) of −1.28 (95% CI: −1.96 to −0.61; *P* < 0.00001). This magnitude of improvement approached the minimal clinically important difference (MCID) for the temporomandibular joint pain scale—commonly defined as 1.0–1.5 points on the visual analogue scale (VAS)—suggesting a clinically meaningful therapeutic effect (49). The observed analgesic response aligns with established biological and biomechanical mechanisms of ESWT, including: (i) mechanical and cavitation effects—shock waves induce controlled microtrauma that stimulates tissue regeneration and enhances local microcirculation; (ii) anti-inflammatory modulation—downregulation of pro-inflammatory cytokines (e.g., TNF-α, IL-1β) and attenuation of peripheral nociceptor sensitization; and (iii) myofascial relaxation—reduction of muscular hypertonicity and restoration of viscoelastic properties in masticatory and periarticular tissues. Functionally, maximum mouth opening (MMO) improved by a mean of 3.76 mm, exceeding the reported MCID of 2–3 mm for TMD-related functional recovery. However, substantial statistical heterogeneity was evident across studies for both pain (I^2^ = 95%) and MMO (I^2^ = 97%), likely attributable to variations in outcome assessment protocols, ESWT parameter selection (e.g., energy flux density, pulse frequency, total number of shocks), and baseline disease severity (50, 51). Such heterogeneity necessitates cautious interpretation of pooled estimates; the observed treatment effects—particularly in pain and mandibular range of motion—are likely influenced by an interplay of clinical, technical, and methodological factors. Consequently, while the overall direction of effect supports the general feasibility of ESWT as a non-invasive adjunctive intervention, its magnitude may vary depending on treatment protocol optimization, follow-up duration, and patient-specific characteristics (e.g., TMD subtype, chronicity, comorbidities) (52). In contrast, no statistically significant improvement was detected in the functional disability index (DI) (MD = −0.05; 95% CI: −0.13 to 0.03; *P* = 0.22), and the observed change fell below the accepted threshold for clinical relevance. This underscores the importance of distinguishing statistical significance from clinical meaningfulness: although pain relief approached the MCID, DI changes were neither statistically nor clinically meaningful. Publication bias was assessed using both Egger's and Begg's tests; no evidence of small-study effects was found for the primary pain outcome. This meta-analysis synthesized data from 18 randomized controlled trials and provides moderate-to-high certainty evidence that ESWT yields clinically relevant reductions in pain and improvements in MMO among patients with TMD. The consistency of these effects across core patient-centered outcomes strengthens the rationale for integrating ESWT into multimodal, non-pharmacologic management strategies. Nevertheless, mechanistic uncertainties persist regarding the precise anatomical and pathophysiological pathways linking ESWT to symptom resolution—particularly in heterogeneous presentations involving joint derangement vs. myofascial pain. Resolving this gap requires rigorous, hypothesis-driven research examining how ESWT interacts with the unique biomechanical and neuroinflammatory milieu of the temporomandibular joint and associated musculature (53, 54).

The subgroup analyses reveal a critical consensus: ESWT consistently alleviates pain and improves MMO across diverse clinical scenarios, with treatment duration recognized as a key moderating factor. Longer intervention courses (≥4 weeks) yielded stronger, more consistent analgesic effects accompanied by significantly reduced heterogeneity, whereas shorter regimens (≤2 weeks) resulted in weaker and more unstable responses. No consistent moderating effects were detected for gender distribution, baseline disease severity, or concurrent adjuvant treatments (55). The lack of significant gender-related modulation initially indicates that the therapeutic benefits of ESWT may be generalizable across diverse patient populations, underscoring its potential as a flexible intervention that can be implemented in varied clinical settings without compromising effectiveness (56). The substantial statistical heterogeneity (I^2^ > 90% for most outcomes) necessitates a cautious and nuanced interpretation of the pooled results. The failure of subgroup analyses to fully explain this variability suggests that observed differences are more likely driven by subtle methodological and clinical variations rather than the predefined high-level factors. Such heterogeneity may stem from a combination of factors, including discrepancies in ESWT energy flux density, pulse frequency, total impulse count, heterogeneous comparator interventions (sham, physical therapy, medication, or no treatment), and inherent phenotypic diversity among TMD patients (56). Consequently, although the direction of therapeutic effect is uniformly favorable, the high heterogeneity implies that the exact magnitude of benefit conferred by ESWT is context-dependent. This underscores that the present findings represent a robust overall trend rather than a fixed effect size, and emphasizes the critical need for standardized reporting in future research to better define optimal application parameters.Notably, the directional patterns of certain trends warrant mechanistic exploration. The more stable efficacy observed in long-term follow-up subgroups may reflect time-dependent tissue remodeling and functional adaptation, although this hypothesis requires validation through long-term prospective cohort studies (57). Similarly, the tendency toward superior efficacy in chronic TMD is consistent with biomechanical principles: persistent myofascial hypertonia and joint dyskinesia respond more favorably to mechanical stimulation than acute inflammatory conditions, whereas short-term ESWT interventions act primarily by modulating nociceptive signaling.

The pathophysiological cascade in TMD involves a self-perpetuating cycle: initial masticatory muscle overload or articular disc displacement disrupts mandibular kinematics, triggering compensatory muscular hyperactivity, elevated joint friction, and local inflammatory infiltration (58). Such mechanical dysfunction further promotes neurogenic inflammation and nociceptor sensitization, ultimately leading to pain-mediated motion restriction, diminished maximal mouth opening, and progressive functional impairment. The therapeutic efficacy of ESWT arises from its capacity to interrupt this pathological cycle through integrated mechanical, biological, and neuromodulatory pathways (59). By delivering controlled mechanical energy to targeted tissues, ESWT enhances local microcirculation, accelerates the removal of proinflammatory mediators, and stimulates fibroblast proliferation and collagen remodeling, thereby restoring elasticity to periarticular soft tissues. For analgesic effects, shock wave-induced sensory input modulates pain processing via the gate control theory, reducing the central transmission of nociceptive signals (60). Concurrently, cavitation-related effects improve tissue perfusion and alleviate interstitial edema, further attenuating peripheral sensitization. This mechanistic framework explains the prominent advantage of ESWT in providing rapid symptomatic and functional recovery, similar to well-established physical interventions for musculoskeletal disorders. Nevertheless, it also reveals an inherent limitation: as a noninvasive biomechanical modulator, ESWT cannot directly reverse severe structural lesions in chronic TMD, including advanced disc displacement, bony remodeling, or irreversible fibrous adhesions. Instead, it acts predominantly by breaking the vicious pain-dysfunction cycle rather than repairing core anatomical defects.

The translation of ESWT's symptomatic efficacy into clinical practice thus necessitates strategic integration within a broader therapeutic paradigm (61). Its profound value lies not as a monotherapy aimed at structural restoration, but as a catalyst for functional engagement during the critical phases of TMD rehabilitation (62, 63). By effectively reducing pain inhibition and improving mandibular mobility, ESWT creates a vital window of opportunity to initiate and sustain mechanoactive interventions—including masticatory muscle training, joint mobilization, and occlusal management—which are indispensable for restoring physiological kinematics and preventing symptom recurrence (64). This synergistic relationship explains why combined ESWT and rehabilitation regimens frequently outperform either approach in isolation. Furthermore, ESWT's favorable safety profile, characterized predominantly by mild and transient local erythema or temporary discomfort, positions it as a low-risk adjunct in contexts where pharmacotherapy carries side effects or invasive interventions are premature or contraindicated. However, the persistent heterogeneity observed across trials, unmitigated even by rigorous subgroup analyses, serves as a potent reminder that ESWT's application is not universally algorithmic. Its efficacy is intrinsically context-dependent, modulated by variables such as TMD subtype (muscular, articular, or mixed), baseline functional deficits, and individual pain sensitivity, demanding a precision-based approach rather than protocolized uniformity (65). Current evidence remains limited by unresolved heterogeneity, insufficient long-term data, and geographic concentration of study populations. Inconsistencies in outcome measurement, ESWT device parameters, and sample characteristics further complicate cross-study comparisons. To advance ESWT's clinical translation for TMD, future studies must establish standardized treatment protocols using validated energy and dosage parameters, while prioritizing longitudinal multicenter designs that integrate quantitative functional assessments and patient-reported outcomes (66). This integrated approach will ultimately clarify ESWT's role in delaying structural progression and inform precision rehabilitation strategies for personalized TMD care.

This meta-analysis has several advantages: It is the first comprehensive review of 18 randomized controlled trials (RCTs), involving 1,150 participants, aiming to assess the effects of electrical stimulation therapy (ESWT) on pain, maximum mouth opening, and functional impairment in patients with temporomandibular joint disorders (TMD). The study strictly followed the guidelines of the Cochrane Handbook, conducted rigorous data extraction, bias risk assessment, sensitivity analysis, and subgroup analysis, and did not find significant publication bias in the main outcome indicators. However, it must be acknowledged that it has several limitations. The high heterogeneity limited the accuracy of the pooled estimates, partly due to differences in ESWT parameters, control intervention measures, and TMD subtypes. Most of the included RCTs were single-center studies conducted in China, which may limit the generalizability of the research results to other populations and healthcare systems. Due to the lack of adequate reporting on the blind randomization and patient/medical staff blinding, the risk of bias is often unclear, so the overall certainty of the evidence is at a moderate level rather than high. The study did not find significant improvement in the functional impairment index, suggesting that ESWT alone may not fully restore the comprehensive function of the jaw joint. Moreover, long-term follow-up data are insufficient for meta-analysis. Therefore, future international, multicenter randomized controlled trials are needed, using standardized protocols, adequate blinding, and extended follow-up periods.

## 5 Conclusion

In summary, our meta-analysis confirms that ESWT has a definitive therapeutic effect in alleviating pain symptoms (assessed via VAS) and improving mouth opening function (measured by MMO) in patients with TMD, with favorable short-term safety profiles. As such, ESWT can be considered one of the preferred non-invasive therapeutic options for TMD management. However, its effects on the temporomandibular joint dysfunction index (DI) remain to be verified by additional high-quality studies. Moving forward, optimizing intervention protocols, supplementing long-term follow-up data, and strengthening multi-disciplinary collaboration will further unlock ESWT's value in TMD management, ultimately providing patients with more precise and comprehensive therapeutic options.

## Data Availability

The original contributions presented in the study are included in the article/Supplementary Material, further inquiries can be directed to the corresponding author/s.
