# Supplementary material for: Extracorporeal shock wave therapy for temporomandibular disorders: efficacy on pain, maximal mouth opening, and dysfunction—a meta-analysis of randomized controlled trials
Source: Front Rehabil Sci. 2026 Jul 9;7:1883205. doi: 10.3389/fresc.2026.1883205 (PMC13391523; doi:10.3389/fresc.2026.1883205)
Supplement: Supplementary file 1 [file Table1.pdf]

Extracorporeal shock wave therapy for temporomandibular disorders: efficacy on pain, maximal mouth opening, and dysfunction—a meta-analysis of randomized controlled trials

Appendix 1 for specific search strategies

Database	Search Strategy	Number of articles
Pub Med	Search: (("Extracorporeal Shockwave Therapy"[Mesh]) OR (((((((((((((((Extracorporeal Shockwave Therapy) OR (Extracorporeal Shockwave Therapies)) OR (Shockwave Therapies, Extracorporeal)) OR (Shockwave Therapy, Extracorporeal)) OR (Therapy, Extracorporeal Shockwave)) OR (Extracorporeal Shock Wave Therapy)) OR (Shock Wave Therapy)) OR (Shock Wave Therapies Therapy)) OR (Shock Wave)) OR (Extracorporeal High-Intensity Focused Ultrasound Therapy)) OR (Extracorporeal High Intensity Focused Ultrasound Therapy)) OR (High-Intensity Focused Ultrasound Therapy)) OR (High Intensity Focused Ultrasound Therapy)) OR (HIFU Therapy)) OR (HIFU Therapies)) OR (Therapy, HIFU))) AND (("Temporomandibular Joint Disorders"[Mesh]) OR (((((((((((((((((((((((temporomandibular disorders) OR (Disorders, Temporomandibular Joint)) OR (Disorder, Temporomandibular Joint)) OR (Joint Disorders, Temporomandibular)) OR (Joint Disorder, Temporomandibular)) OR (Temporomandibular Joint Disorder)) OR (Temporomandibular Joint Diseases)) OR (Diseases, Temporomandibular Joint)) OR (Disease, Temporomandibular Joint)) OR (Joint Diseases, Temporomandibular)) OR (Joint Disease, Temporomandibular)) OR (Temporomandibular Joint Disease)) OR (TMJ Diseases)) OR (Diseases, TMJ)) OR (Disease, TMJ)) OR (TMJ Disease)) OR (Temporomandibular Disorders)) OR (Disorders, Temporomandibular)) OR (Disorder, Temporomandibular)) OR (Temporomandibular Disorder)) OR (TMJ Disorders)) OR (Disorders, TMJ)) OR (Disorder, TMJ)) OR (TMJ Disorder)))	13
Web of Science	Refine results for "craniomandibular disorders" OR "craniomandibular joint syndrome'"OR "temporomandibular disorder" OR "temporomandibular dysfunction" OR "temporomandibular joint	21

	disease" OR "temporomandibular joint diseases" OR "temporomandibular joint disorders" OR "temporomandibular joint dysfunction" OR "temporomandibular joint dysfunction syndrome" (Abstract) AND "Extracorporeal Shockwave Therapies"" OR "Shockwave Therapies, Extracorporeal" OR " Shockwave Therapy, Extracorporeal" OR "Therapy, Extracorporeal Shockwave" OR " Extracorporeal Shock Wave Therapy" OR "Shock Wave Therapy" OR "Shock Wave Therapies" OR "Therapy, Shock Wave" OR "Extracorporeal High-Intensity Focused Ultrasound Therapy" (Abstract)	
Cochrane Library	ti,ab,kw OR (Shock Wave Therapies):ti,ab,kw OR (Shockwave Therapies, Extracorporeal):ti,ab,kw OR (Therapy, Extracorporeal Shockwave):ti,ab,kw OR (Extracorporeal Shock Wave Therapy)ti,ab,kw OR (Shock Wave Therapy):ti,ab,kw OR (Therapy, Shock Wave):ti,ab,kw OR (Extracorporeal Shockwave Therapies):ti,ab,kw OR (Shockwave Therapy, Extracorporeal)ti,ab,kw OR (Extracorporeal High-Intensity Focused Ultrasound Therapy):ti,ab,kw OR (HIFU Therapy):ti,ab,kw OR (High Intensity Focused Ultrasound Therapy):ti,ab,kw OR (Extracorporeal High Intensity Focused Ultrasound Therapy)ti,ab,kw OR (High-Intensity Focused Ultrasound Therapy):ti,ab,kw OR (Therapy, HIFU):ti,ab,kw OR (HIFU Therapies) and ti,ab,kw OR (Disorder, TMJ):ti,ab,kw OR (Disease, TMJ):ti,ab,kw OR (Disorders, Temporomandibular Joint):ti,ab,kw OR (Joint Disease, Temporomandibular)ti,ab,kw OR (Temporomandibular Joint Disorder):ti,ab,kw OR (TMJ Disorders):ti,ab,kw OR (Temporomandibular Disorder):ti,ab,kw OR (Diseases, TMJ)ti,ab,kw OR (Diseases, TMJ):ti,ab,kw OR (Disorders, Temporomandibular):ti,ab,kw OR (TMJ Diseases):ti,ab,kw OR (Diseases, Temporomandibular Joint)ti,ab,kw OR (Disorder, Temporomandibular):ti,ab,kw OR (Disorder, Temporomandibular Joint):ti,ab,kw OR (TMJ disease):ti,ab,kw OR (Temporomandibular Joint Disease)ti,ab,kw OR (Temporomandibular Disorders):ti,ab,kw OR (Joint Disorders, Temporomandibular):ti,ab,kw OR (Temporomandibular Joint Diseases):ti,ab,kw OR (Disorders, TMJ)	11

Embase	'temporomandibular joint disorder'/exp and 'costen syndrome'/exp OR 'costen syndrome' OR 'craniomandibular disorders'/exp OR 'craniomandibular disorders' OR 'craniomandibular joint syndrome'/exp OR 'craniomandibular joint syndrome' OR 'temporomandibular disorder'/exp OR 'temporomandibular disorder' OR 'temporomandibular dysfunction'/exp OR 'temporomandibular dysfunction' OR 'temporomandibular joint disease'/exp OR 'temporomandibular joint disease' OR 'temporomandibular joint diseases'/exp OR 'temporomandibular joint diseases' OR 'temporomandibular joint disorders'/exp OR 'temporomandibular joint disorders' OR 'temporomandibular joint dysfunction'/exp OR 'temporomandibular joint dysfunction' OR 'temporomandibular joint dysfunction syndrome'/exp OR 'temporomandibular joint dysfunction syndrome' OR 'temporomandibular joint pain'/exp OR 'temporomandibular joint pain' OR 'temporomandibular joint syndrome'/exp OR 'temporomandibular joint syndrome' OR 'temporomandibular joint disorder'/exp OR 'temporomandibular joint disorder'	5
CNKI (China National Knowledge Infrastructure)	English version： (Topic: (“Shock wave therapy”+ “Extracorporeal shock wave therapy”) AND (“Temporomandibular joint disorder syndrome” + “Temporomandibular joint arthritis” + “Costin syndrome” + “Temporomandibular joint disorder “+ “Temporomandibular joint disorder disease”)	23
Wanfang ---Chinese Database	English version： (Topic: (“Shock wave therapy”+ “Extracorporeal shock wave therapy”) AND (“Temporomandibular joint disorder syndrome” + “Temporomandibular joint arthritis” + “Costin syndrome” + “Temporomandibular joint disorder “+ “Temporomandibular joint disorder disease”)	17
VPCS (Weipu website)	English version： (Topic: (“Shock wave therapy”+ “Extracorporeal shock wave therapy”) AND (“Temporomandibular joint disorder syndrome” + “Temporomandibular joint arthritis” + “Costin syndrome” + “Temporomandibular joint disorder “+ “Temporomandibular joint disorder disease”)	20
